# Bioavailability Enhancement of Curcumin by PEG-Based Gastroretentive System: Development and In Vitro Evaluation

**DOI:** 10.3390/pharmaceutics17091166

**Published:** 2025-09-05

**Authors:** Orsolya Csendes, Gábor Vasvári, Ádám Haimhoffer, László Horváth, Monika Béresová, Attila Bényei, Ildikó Bácskay, Pálma Fehér, Zoltán Ujhelyi, Dániel Nemes

**Affiliations:** 1 Department of Pharmaceutical Technology, Faculty of Pharmacy, University of Debrecen, Rex Ferenc St. 1, H-4002 Debrecen, Hungary; csendes.orsolya@pharm.unideb.hu (O.C.); haimhoffer.adam@euipar.unideb.hu (Á.H.); bacskay.ildiko@pharm.unideb.hu (I.B.); feher.palma@pharm.unideb.hu (P.F.); 2 Doctoral School of Pharmaceutical Sciences, University of Debrecen, H-4032 Debrecen, Hungary; 3 Department of Pharmaceutical Surveillance and Economics, Faculty of Pharmacy, University of Debrecen, Rex Ferenc St. 1, H-4002 Debrecen, Hungary; horvath.laszlo@pharm.unideb.hu; 4 Department of Medical Imaging, Faculty of Medicine, University of Debrecen, H-4032 Debrecen, Hungary; beres.monika@med.unideb.hu; 5 Department of Physical Chemistry, University of Debrecen, Egyetem Sq. 1., H-4032 Debrecen, Hungary; benyei.attila@science.unideb.hu; 6 Institute of Healthcare Industry, University of Debrecen, Rex Ferenc St. 1, H-4002 Debrecen, Hungary; 7 Department of Industrial Pharmaceutical Technology, Faculty of Pharmacy, University of Debrecen, Rex Ferenc St. 1, H-4002 Debrecen, Hungary; ujhelyi.zoltan@pharm.unideb.hu

**Keywords:** curcumin, self-emulsifying system, gastroretention, bioavailability, melt-foaming, extended release

## Abstract

**Background/Objectives:** Increasing the bioavailability of poorly absorbed drugs is a continuous challenge in modern pharmaceutical technology. This is due to the problematic nature of BCS class IV active pharmaceutical ingredients: these drugs possess poor solubility and membrane permeability. Moreover, many undergo immediate efflux and/or rapid systemic metabolism after absorption. This project aimed to improve the bioavailability of BCS class IV drugs by formulating gastroretentive self-emulsifying systems using curcumin as a model drug. **Methods:** The base of the systems was created by melting emulsifying agents, dissolution retardants, and PEGs together. Curcumin was added after the mixture was cooled slightly. Aqueous dispersions of several compositions were characterized by dynamic light scattering. After screening these results, the viscosities of the selected formulations were evaluated. Dissolution retardants were selected and added to the most superior samples, and their dissolution profiles were compared. Gastroretention of the final formulation was achieved by dispersing air in the molten system through melt foaming; internal structure was assessed by microCT, and physicochemical properties by PXRD and DSC. Cytotoxicity was measured in Caco-2 cells using MTT and Neutral Red assays, and transcellular transport was also studied. **Results:** Based on these results, a homogeneous gastric floating system was developed. We observed an advantageous cytotoxic profile and increased bioavailability. **Conclusions:** Overall, we were able to create a self-emulsifying gastroretentive formulation displaying extended release and gastric retention with a low amount of cost-efficient excipients.

## 1. Introduction

Oral drug delivery is the most frequently used method of drug administration worldwide. The main reasons for this are easy administration and consequently higher patient compliance compared to other drug delivery routes. Although oral delivery is widespread, it has certain limitations. One of the most significant factors is the variability in absorption from the gastrointestinal tract. Many factors influence the absorption process, such as age, sex, diet, genetics, and various diseases. Another important factor is gastric emptying. Some active pharmaceutical ingredients are best absorbed in the upper parts of the gastrointestinal tract, namely, the stomach and duodenum. Moreover, for drugs that are best absorbed in the stomach, short gastric emptying results in incomplete drug absorption from the delivery system and a decrease in effect [1,2]. The excipients used can also play an important role in the formulation process, as they can modify the liberation, solubility, absorption, or transit time of the applied active pharmaceutical ingredient [3].

The biopharmaceutical classification system (BCS) is a well-established method for classifying active pharmaceutical ingredients (APIs) based on their water solubility and lipid permeability [4], which are the two most important properties that play a crucial role in the bioavailability of a given molecule. Class I drugs are highly soluble and permeable, making them ideal for oral administration. (Such drugs are paracetamol, theophylline, etc.) Class II members (e.g., ketoconazole and carbamazepine) have high permeability but poor water solubility. In this case, the limiting factor was the slow solvation rate. In the case of class III drugs (such as metformin or acyclovir), the water solubility is high, but they do not permeate easily through biological barriers, which means that the main limiting factor is the poor permeation rate, despite the quick solvation process. Drugs belonging to class IV have both poor solubility and permeability. Notable examples include furosemide and ritonavir. Examining the on-market and pipeline drugs, it can be noted that there is an increasing number of APIs with low solubility [5]. Consequently, class IV drugs are the focus of the development of new, innovative drug delivery systems [6].

Curcumin (CUR) was selected as the model active ingredient in this study. This is due to the fact that curcumin belongs to the BCS class IV, as its water solubility is only 5.75 µg/mL [7] and its permeability is approximately 3 × 10^−6^ cm/s [8]. Additionally, curcumin is a potent antioxidant and free radical scavenger, which is responsible for its anti-inflammatory, antidiabetic [9], antimutagenic, and antimicrobial effects [10,11]. To improve curcumin bioavailability [12], we focused on enhancing its solubility in water. The scientific literature offers multiple examples of this, such as encapsulation in cyclodextrin complexes [13], salification [14], esterification [15], or the addition of surface-active agents, which was our chosen method [16].

Solid self-emulsifying drug delivery systems (SEDDSs) combine the benefits of solid dosage forms and self-emulsifying systems. Compared to liquid SEDDSs, solid systems possess more advantageous microbiological and thermodynamic stability [17], and in contrast to conventional solid forms, they offer increased bioavailability [18]. In addition to the API, SEDDSs are composed of a lipid-like part (natural or synthetic oils) and one or more surfactants and co-surfactants. Upon contact with water-based media (usually gastric juice) and slight agitation (motility of the gastrointestinal tract), these formulations spontaneously form a finely dispersed O/W emulsion [19,20,21].

Lipid-based drug carrier systems can be sorted into four major groups based on their composition, as previously researched by Pouton et al. [22] The hydrophilicity of the systems increases with their number of respective groups. Type I systems consist entirely of glycerides, limiting their dispersion in the gastrointestinal system. The particle size distribution of the dispersion was coarse. Systems belonging to type II typically contain a lipid phase (40–80%) together with a hydrophobic surfactant capable of forming W/O emulsions (20–60%, hydrophilic–lipophilic balance (HLB) lower than 12). The resulting dispersion was a colloidal system with particle sizes ranging from 100 to 250 nm. Type III formulations can be further classified into two categories: IIIA and IIIB. IIIA systems contain 40–80% lipid phase with 20–40% O/W surfactant (HLB greater than 11) together with 0–40% hydrophilic co-solvents. The particles of the formed emulsion typically fall into the 100–250 nm range. Type IIIB systems have low lipid content (≤20%), whereas the amount of surfactant (20–50%) and co-solvents (20–50%) is increased. This leads to increased hydrophilicity, with a droplet size range of 50–100 nm. Type IV systems are the most hydrophilic, consisting solely of HLB ≤ 12 surfactants (0–20%), HLB ≥ 11 surfactants (30–80%), and hydrophilic co-solvents (0–50%). The particles in their emulsions are less than 50 nm in diameter [23,24,25].

The emulsions formed by these self-emulsifying systems can be further characterized as macro-, micro-, and nanoemulsions. Macroemulsions have a particle size range of 1–100 µm and large polydispersity, which causes them to appear opaque. These systems are thermodynamically unstable and exhibit weak kinetic stability [20,26,27]. Self-microemulsifying drug delivery systems (SMEDDSs) form an isotropic mixture that is thermodynamically stable and appears as a transparent or translucent liquid. The particle size is usually within the 10–100 nm range, and they have low polydispersity [26,27]. Finally, self-nanoemulsifying drug delivery systems (SNEDDSs) form emulsion droplets with a diameter of 20–500 nm and low polydispersity. Emulsions are thermodynamically unstable but kinetically stable. The emulsion typically has a clear appearance [16,26,28].

By extending the release of the API from the product, the absorption and effect of the drug are increased. Gastroretention is the ability to resist gastric motility, thus remaining inside the stomach and ensuring an extended drug release into the gastric juice [29,30,31]. This is highly advantageous when a locally high concentration and residual time is needed, such as *Helicobacter pylori* eradication, or in the case of APIs that have superior absorption in an acidic environment compared to other parts of the gastrointestinal tract, such as nilotinib [32,33,34]. Gastroretention can be achieved via multiple methods, such as low-density floating, mucoadhesive, swelling, and extending or high-density drug delivery systems, and in many cases, combinations of these options [35,36,37].

The combination of gastroretentive and self-emulsifying systems offers longer gastric retention time and ensures that the API has increased dissolution in the gastric juice, resulting in higher bioavailability of the drug [20]. In this study, our goal was to formulate low-density floating systems, which we planned to achieve using the melt-foaming method. A schematic of our in-house built apparatus with an agitator can be seen in [38]. The process of melt foaming was previously investigated in our department, and the findings were published in multiple papers. The developed formulation is unconventional considering its composition: usually solid SEDDSs are made by blending liquid SEDDSs with a solid adsorbent material, and thus, the adsorbent is necessary to achieve a solid system. In our study, we aimed to produce a self-emulsifying system that is inherently solid, without the addition of adsorbents, which is highly favorable in the case of direct compression. Additionally, the composition we designed was tailored to provide prolonged release of a BCS class IV molecule, namely curcumin. The current study demonstrates that our gastroretentive system is suitable for the release of hydrophobic drugs, with improved in vitro permeability.

## 2. Materials and Methods

### 2.1. Materials

When selecting excipients, safe oral administration and low toxicity were the most important parameters. The selected materials are considered “Generally Regarded As Safe” (GRAS) by the FDA, and their previous safe use has been documented by multiple research groups.

Glycerol distearate (type I) (GS; Precirol ATO 5 [39,40]), and Gelucire 44/14 [40] and 48/16 [41] were gifts of Gattefossé (Lyon, France) through Azelis Hungary Ltd. (Budapest, Hungary). Curcumin (CUR) (synthetic, >97.0%), Ph. Eur. grade stearic acid (type 50) (SA [42]), polyethylene glycol 4000 (PEG 4000 [42]), and PEG 6000 [41] were purchased from Molar Chemicals Ltd. (Halásztelek, Hungary). Kolliphor RH40 [43], stearylamine (≥99%), (SM), and other reagents (analytical grade) were purchased from Merck KGaA (Darmstadt, Germany). All materials have well-established use in cases of SEDDS development.

### 2.2. Preparation of Self-Emulsifying Systems

Curcumin and other components were measured using an analytical balance. All components, except CUR, were heated to 70 °C and mixed to form a homogenous melt. Then, CUR was added and dissolved by mixing on a heating magnetic stirrer. The amounts of each component are listed in Table 1. The concentrations and components were based on literature examples and general recommendations for SEDDSs [22,26,44,45].

### 2.3. Dynamic Light Scattering (DLS) Measurements for Droplet Size Distribution

An amount of 0.1 g of each composition was dispersed in 90 mL of artificial gastric juice (prepared according to Ph. Eur.). Fragmentation using a spatula spoon aided the rapid disintegration and dissolution of the sample. After the total dissolution of the samples, the dispersion was pipetted into polystyrene cuvettes and immediately measured. Three parallel measurements were performed using a Malvern Nano-ZSP Zetasizer instrument (Malvern Instruments, Malvern, UK).

### 2.4. Apparent Viscosity Measurement of the Molten SEDDS Containing Curcumin

The apparent viscosity of the samples was studied using a Rheolab QC rotational rheometer (Anton Paar GmbH, Graz, Austria) with a CC27 concentric cylinder system and a constant temperature maintained using a rheometer-controlled water bath. Three parallel measurements were performed at a constant shear rate of 1000 RPM. After adding the solid samples into the measuring cup, they were heated to 61 °C, and the measurement was started. The viscosity curves were recorded using the manufacturer-developed RheoPlus 2.6x software between 61 °C and 45 °C.

### 2.5. Melt Foaming

Melt foaming was carried out according to our previously developed and published technology to create molten dispersions at atmospheric pressure using benchtop equipment [38,42]. Forty grams of composition S14 with 10% SM was prepared as described in Section 2.2. The hot dispersion was loaded into a jacketed cylinder at 70 °C. The molten material was cooled to 53 °C and foamed at an agitator speed of 1500 rpm. The foamed dispersion was filled into cavities with a bullet shape (V: 1.027 mL) of a metal mold, from which they were removed after cooling.

### 2.6. PXRD Study

Paraffin oil was used to fix the pulverized samples on a MiTeGen MicroMeshes (MiTeGen LLC., Ithaca, NY, USA) sample holder. A Bruker-D8 Venture diffractometer (Bruker, Billerica, MA, USA) equipped with INCOATEC IμS 3.0 dual (Cu and Mo) sealed tube microsources (Incoatec GmbH, Geesthacht, Germany) and a Photon 200 Charge-integrating Pixel Array detector was used for data collection with Debye–Scherrer geometry. For all measurements, room temperature and CuKα (λ = 1.54178 Å) radiation were used. Data collection was performed by 360° phi scans for 2 min at theta = 12, 24, and 36°; omega = −6°; chi 0° with 150 mm sample-detector distance. Images were created in the phi scan mode and merged. Data evaluation was carried out using APEX3 (Version 2018.7-2, BrukerAXS Inc., Madison, WI, USA) and DiffracEva software (Version 4.2.2.3, BrukerAXS Inc., Madison, WI, USA), respectively.

### 2.7. Differential Scanning Calorimetry

A DSC 300 Caliris Supreme (Netzsch-Gerätebau GmbH, Selb, Bavaria, Germany) instrument was used for the experiments. The samples (10 ± 1 mg) were encapsulated into aluminum pans (V = 40 µL), which were then hermetically sealed and manually pierced. DSC curves were recorded from 20 °C (293 K) to 250 °C (523 K) with a heating rate of 10 K min^−1^ under a protective nitrogen atmosphere (99.996% purity, 20 cm^3^ min^−1^ flow rate). An empty, sealed, and pierced aluminum pan (m = 51.222 mg) was used as a reference. Data collection and evaluation of the curves were performed using the Proteus^®^ 9.x Analysis software (Netzsch-Gerätebau GmbH, Selb, Bavaria, Germany).

### 2.8. In Vitro Drug Dissolution

An ERWEKA DT 626 dissolution tester (ERWEKA GmbH, Langen, Hessen, Germany) assembled for the rotating paddle method with a paddle speed of 75 RPM was used for the three parallel samples of the two selected formulations. Hydrochloric acid medium (900 mL, pH 1.2) was loaded into the dissolution vessels and maintained at 37 °C during the tests [38]. Ultrasound was applied to remove the dissolved air. Samples (V: 3.00 mL) were taken at 15 min; 30 min; and 1, 2, 3, 4, and 5 h and filtered through 0.22 μm PES membrane syringe filters. After dilution with 96% *(v*/*v)* ethanol, the amount of dissolved CUR was measured using a Shimadzu UV-1900 UV/VIS spectrophotometer (Shimadzu Corporation, Kyoto, Japan) at 428 nm. During the tests, flotation was visually inspected and followed. The test samples S14 SM10% and S15 SM10% were filled into cavities with a bullet shape (V: 1.027 mL) of a metal mold, which was later used for dissolution studies to ensure equal quantities during dissolution.

Mathematical analysis was used to determine the dissolution profiles of the two compositions. The measured concentrations of the released curcumin were fitted to first-order, zero-order, and Korsmeyer–Peppas model equations. The determination coefficients of the obtained linear equations were evaluated, and release kinetics with a similarity index more than 0.9 were accepted as correlating with a certain model. The following equations were used for the evaluation:

For the zero-order kinetics model:

(1) $$Q=Q_{0}+k_{0}t$$

For the first-order kinetics model:

(2) $$Q_{t}=Q_{0}\times e^{{-k}_{1}\times t}$$

For the Korsmeyer–Peppas kinetics:

(3) $$\frac{Q_{t}}{Q_{\infty }}=k_{kp}\times t^{n}$$

where *Q* is the amount of API released at time *t*, *Q*_0_ is the initial amount of API, *Q_t_* is the amount of remaining API at time *t*, and *Q_t_*/*Q*_∞_ is the fraction of API released by time *t*. *k*_0_, *k*_1_, and *k_kp_* are the kinetic constants for the zero-order, first-order, and Korsmeyer–Peppas models, respectively, whereas *n* is the release exponent [38].

### 2.9. Microtomography and Size Distribution of Foam Bubbles

The internal structure of the bullet-shaped sample was assessed using a compact desktop micro-CT system, SkyScan 1272 (Bruker, Billerica, MA, USA). The image pixel size was 5 µm with a 2688 × 4032 matrix size, without any further correction, at 50 kV and 200 µA. The cross-sectional images were reconstructed from tomography projection images using the SkyScan NRecon package (version: 2.0.4.2) with post-alignment, beam-hardening correction, ring artifact correction, and smoothing. DICOM and BPM formats were used for the images.

CTAn software (version: 1.18.8.0 64-bit) was used for the 3D ROI analyses. Thresholding, region of interest (ROI) shrink-wrap, Relode Image, and 3D analysis plugins were applied. After removing the background, the bubbles inside the sample were automatically segmented (threshold based) from the ROI area (global gray scale 0–255, bubble 0–80). The 3D visualization was performed using DataViewer (version: 1.5.6.2 64-bit) and CTVox (version: 3.3.0 r1403 64-bit) software, depicting the ROI region with RGB color coding.

### 2.10. Cell Culturing and Cytotoxicity Assays

The Caco-2 cell line, which is a well-established in vitro model for both intestinal absorption and oral toxicity [46,47], was obtained from the European Collection of Authenticated Cell Cultures (Catalogue number: 86010202 ECACC, UK Health Security Agency, Porton Down, UK, purchased through Merck KGaA, Darmstadt, Germany). Cells were maintained in Dulbecco’s modified Eagle’s medium (Merck KGaA, Darmstadt, Germany) supplemented with 10.0% heat-inactivated fetal bovine serum, 1.0% non-essential amino acid, and penicillin–streptomycin solution at 37 °C in a 5% CO_2_ atmosphere. The passage number of the cells was between 20 and 32.

The cytotoxic effects of composition S14 with 10% SM were tested using MTT and Neutral Red assays. We employed two assays to complement each other, as yellow MTT is reduced to purple formazan crystals through mitochondrial activity, while the Neutral Red stain is taken up by passive diffusion [48]. Test solutions were prepared by dispersing 1 g of the sample in 900.0 mL of Hanks’ balanced salt solution (HBSS). Caco-2 cells were seeded into 96-well plates at a concentration of 1 × 10^4^ cells/well and grown for 7 days. During treatment, the medium was removed, and 100 µL of each test solution was added to six wells for 30 min. After treatment, the cells were washed twice with phosphate-buffered saline (PBS). In the MTT assay, 100 µL of 0.5 mg/mL MTT solution (PBS as solvent) was added to each well. For the Neutral Red assay, 100 µL of 16.6 mg/mL NR solution (cell culture medium as a solvent) was added to each well. The MTT assay was performed after 3 h of incubation, while the Neutral Red assay was performed after 2 h of incubation. The staining solutions were removed, and the cells were completely dissolved in 100 µL of an isopropanol-1 M hydrochloric acid (25:1) mixture. The absorbance of the wells was measured at 565 nm for the MTT assay and 540 nm for the NR assay using empty wells as the background on a Thermo-Fisher Multiskan Go (Thermo-Fisher, Waltham, MA, USA) microplate reader. The control wells, which were treated with HBSS, were considered to have 100% cell viability.

### 2.11. Cellular Transportation Test

Cells were seeded into Corning^®^ Transwell^®^ polycarbonate cell culture inserts (area: 1.12 cm^2^, pore size: 0.4 µm, 250.000 cells/well) (Corning Inc., Corning, NY, USA) and allowed to grow into a monolayer [49]. The growth process was regularly monitored by measuring the transepithelial electrical resistance (TEER) of the monolayer using a Millicell ERS Voltohmmeter (MilliporeSigma, Burlington, MA, USA). After reaching the required 900 Ω*cm^2^ TEER value, permeability tests were performed in triplicate.

Samples of curcumin and curcumin-containing SEDDSs were prepared in Fetal Bovine Serum. The SEDDS sample was created by dispersing 1 g of the SEDDS in 900 mL of FBS. The control sample was prepared by dispersing 35.29 mg of pure curcumin in 900 mL of FBS. The protein fraction was precipitated using acetonitrile at a 2:1 volume ratio (acetonitrile/sample). The solutions obtained were analyzed using a Hitachi LaChrom ELITE HPLC system equipped with a Photodiode Array Detector (Hitachi High-Tech Corp., Tokyo, Japan). An Agilent HC-C18(2) (Agilent Technologies Inc., Santa Clara, CA, USA) reverse-phase column (4.6 × 150 mm, 5 µm, 400 bar) was used for the experiments, heated to and kept at 40 °C. The detector was set at 430 nm. A 4:6 ratio of acetonitrile and 2% acetic acid solution was used as the mobile phase with a flow rate of 1.0 m/min. Data collection, processing, and analysis were performed using EZChrom Elite Software™ version 3.2.0 (Hitachi High-Tech Corp., Tokyo, Japan) from 10 µL of both samples.

## 3. Results

### 3.1. Droplet Sizes of Self-Emulsifying Systems

Using DLS analysis, each original composition was examined in a biorelevant solution. We determined the particle sizes and polydispersity indices of the hydrated emulsion droplets in each solution. The received particle sizes are listed in Table 2.

Compositions with less ideal traits were discarded when selecting candidates for further experiments. These critical traits were the average particle size (z average) and the polydispersity index (PDI). We aimed to create type IV self-nanoemulsifying systems; therefore, the upper limit of particle size was set to 32 nm, whereas the upper limit of PDI was 0.3. Thus, 9 out of 20 samples (S5–S8, S11, S14, S15, S17, and S18) were selected for further viscosity studies, where their respective melting/freezing points were determined.

### 3.2. Apparent Viscosity of Molten Curcumin-Loaded SEDDSs

**Figure 1:**
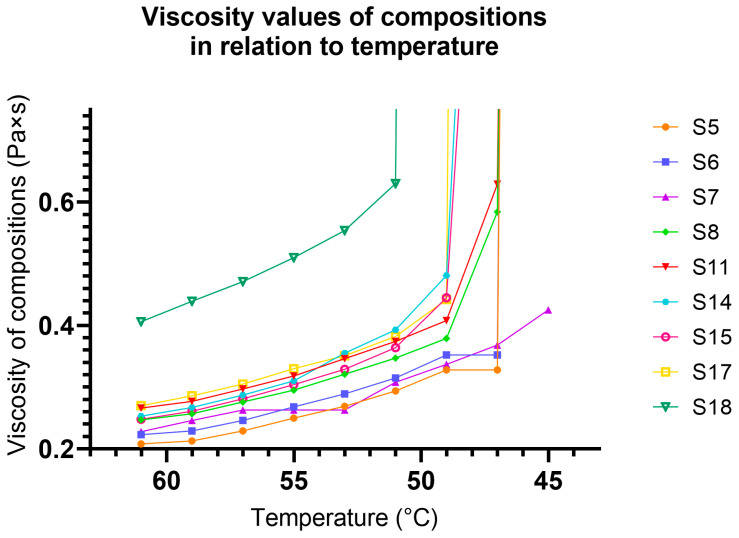
Viscosity curves of selected SEDDS formulations as a function of temperature. The figure also represents the behavior of the particles during solidification.

Based on the DLS measurement results, only the previously selected SEDDSs were used for viscosity measurements. Figure 1 represents the viscosity–temperature curves of the SEDDSs.

As shown in Figure 1, the viscosity of each selected composition was measured on a 61–45 °C spectrum. This informative experiment was conducted to reveal the solidification behavior of the compositions. We found that S7 had a freezing point below 45 °C and an ointment-like texture that lacked the ability to resist deformation forces. On the other hand, S18 had a freezing point of 51 °C, but with a tendency for breaking or chipping. As all compositions had melting points above body temperature, all compositions (S5–S8, S11, S14, S15, S17, and S18) were modified using dissolution retardants.

### 3.3. Effect of Dissolution Retardants on the Droplet Size

Following the viscosity measurement, dissolution retardants were added to the formulation, and their droplet sizes were measured again using DLS. These formulations were prepared using the same method described in Section 2.2. The dissolution retardants stearic acid (SA), glycerol distearate (GS), and stearylamine (SM) were used at a level of 5%, and their addition did not replace any other material; they were added above the original components, which were considered as 95% mass. The results of the droplet size measurements following the addition of dissolution retardants are presented in Table 3.

As expected, droplet sizes increased after the addition of lipophilic dissolution retardants to the mixtures. Comparing the results, we can conclude that SA had the most prominent effect on modifying the particle size of the original nanoemulsions. GS also increased the average droplet sizes (range of 1038–3772 nm), while in the case of 5% SM, the droplet sizes were less than 1 micron for formulations S6 and S7. However, S17, S14, and S18 showed average droplet sizes greater than 2 or 3 microns. Considering the unfavorable effects of GS and SA on particle size, further experiments were conducted only with different concentrations of SM.

### 3.4. Effect of Stearylamine Content

#### 3.4.1. Droplet Size Distribution

We examined compositions (S5–S18) containing 5 and 10% (*m*/*m*) stearylamine. The results are summarized in Table 4.

The z average values indicate that the level of lipophilic dissolution retardants should be optimized in the solid formulations. Interestingly, in the cases of S6, S7, and S17, the droplet sizes increased to 2.5 and 6.3 µm. However, S14 and S15 showed a decrease in the z-average values. Namely, from 2687 nm to 2122 in the case of S14 and from 1203 to 666.6 nm for the composition of S15. Compositions S14 S10% and S15 SM10% had the lowest z-average values among all tested compositions; thus, they were selected for the dissolution tests.

#### 3.4.2. Drug Dissolution

**Figure 2:**
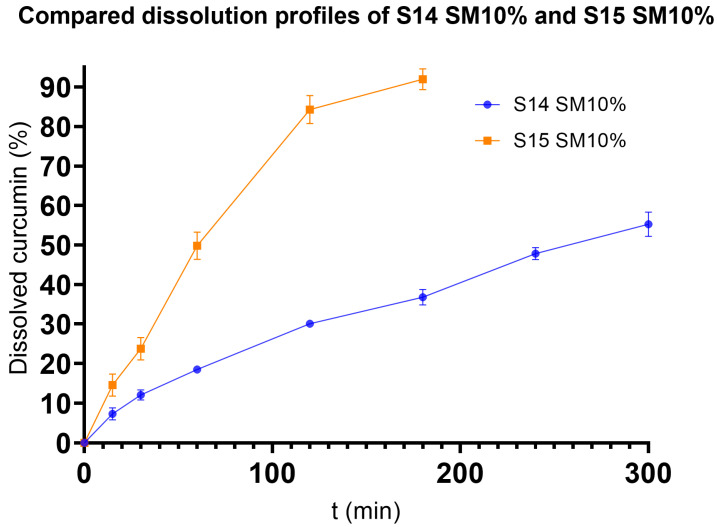
Dissolution test results of S14 and S15 with 10% SM content.

The results of the dissolution tests are shown in Figure 2. Based on the results of the droplet size analysis, the S14 SM10% and S15 SM10% formulations (S14 and S15 containing 10% SM) were subjected to dissolution tests in pH 1.2 hydrochloric acid media. Figure 2 shows the dissolution profiles of both formulations. When examining the release data of S15 SM10%, it is evident that the release of curcumin is too swift, taking only three hours to release nearly 92%.

As shown in Figure 2, in the case of S14 SM10%, during the 5 h dissolution test, only 55% of its curcumin content was released into the dissolution media. The determination coefficients were used to determine the best fit. None of the models fitted the Korsmeyer–Peppas model, since the correlation coefficients were all less than 0.90 (in this model, only the part of the release curve where dissolved API is less than 60% can be used. In the case of S15 SM10%, only three data points had values less than 60%; thus, these were taken into consideration). In contrast, the calculations revealed that the release data of S14 SM10% fitted best to the first-order kinetic model (Table 5) [50,51]. Based on these data, the following experiments were carried out only on S14 SM10%, which was considered the final formulation.

### 3.5. Structural Characterization of S14 SM10% Using PXRD

Using the X-Ray Powder Diffraction technique, we analyzed the diffractograms of the solid S14 SM10% formulation, as well as pure crystalline curcumin, PEG 6000, Gelucire 44/14, Kolliphor RH40, and octadecylamine. The diffractogram of S14 SM10% was compared to all three materials.

**Figure 3:**
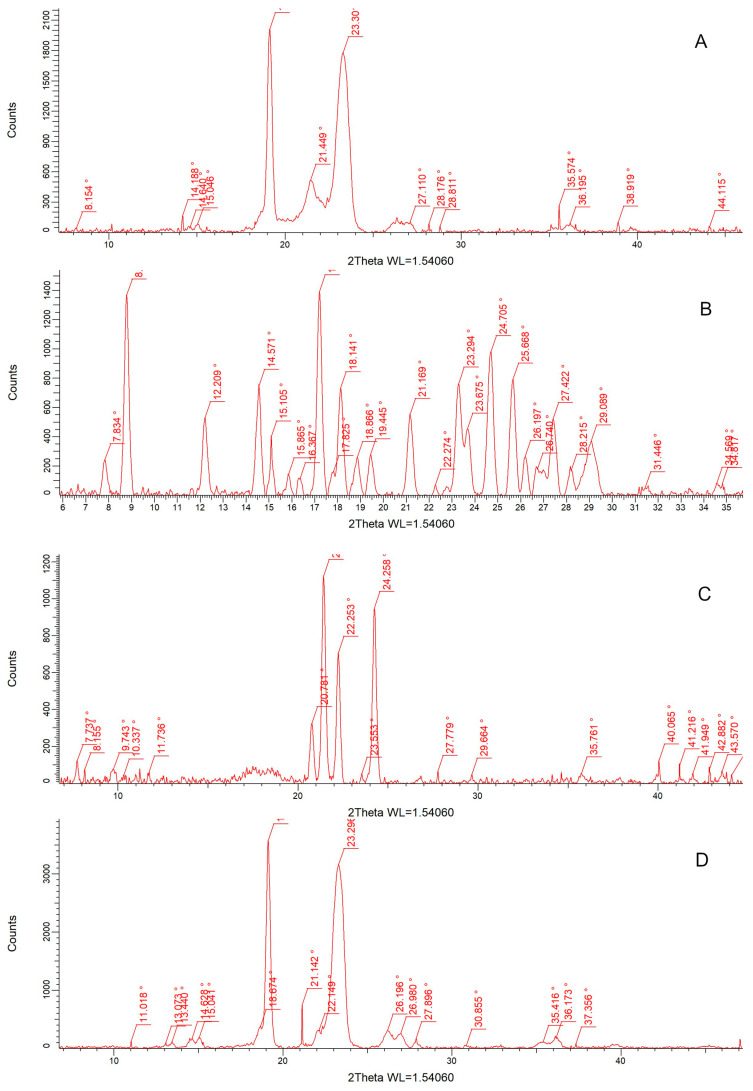

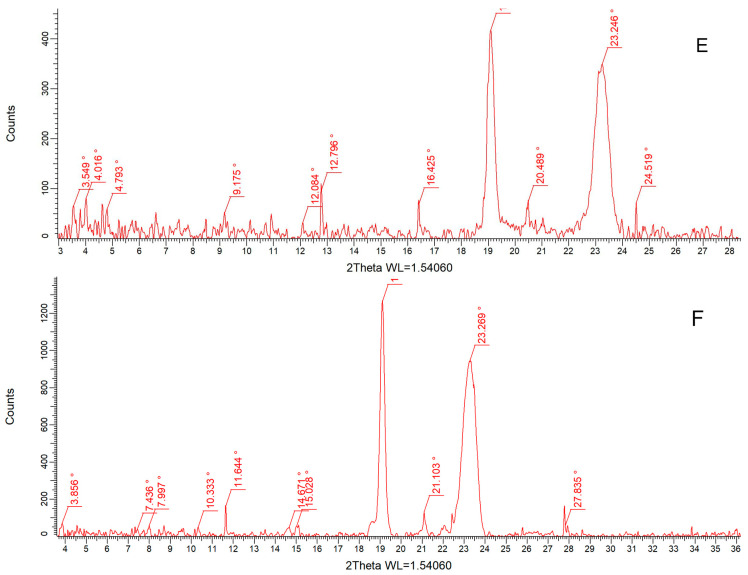
Acquired RTG diffractograms of S14 SM10% (**A**), crystalline curcumin (**B**), octadecylamine (**C**), PEG 6000 (**D**), Kolliphor RH40 (**E**), and Gelucire 44/14 (**F**).

The diffractogram of CUR (Figure 3B) represents the crystalline nature of the API [52]. However, the characteristic peaks of it were not present in the formulation (Figure 3A), indicating that it was dissolved in the Gelucire–PEG matrix. When comparing diffractograms A with C and D, the main peaks of the composition were derived from PEG 6000 as the main excipient, with 2θ values of 23.3° and 19.2°, respectively, and from SM, with a 2θ value of 21.45°.

### 3.6. Results of Foaming Process

After confirming the optimal dissolution of the API in the formulation, we produced a solid system with a lower density than water using the method and parameters described in our previous publications [38,42] and in Section 2.5.

After demolding the solidified products, we gently brushed their surfaces. The cleaned products were measured using an analytical balance, and the density was calculated by knowing the volume of the molding forms. The resulting average density was 0.97 g/cm^3^, with a standard deviation of 0.01, while the initial density of the composition was 1.05 ± 0.03 g/cm^3^. This indicates that the process resulted in a 7.62% decrease in density values.

### 3.7. Microtomographic Assessment of Solid Foam Structure

**Figure 4:**
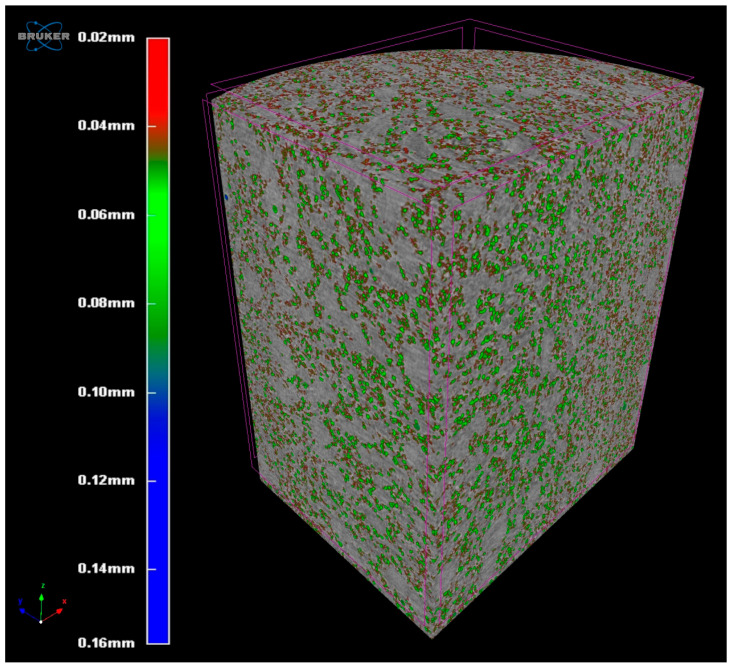
MicroCT-scan imaging of S14 SM10% solid foam structure.

Looking at Figure 4, it can be said that the distribution of the air bubbles in the matrix is homogeneous. Inside the sample, we did not find any larger voids, indicating the uneven distribution of air or quick bubble coalescence during the molding process of the hot-foamed compositions. The scans also confirmed that a closed-cell structure was created. Negligible amounts of voids were found to be open to the environment, and the outer layer of our sample was smooth.

**Figure 5:**
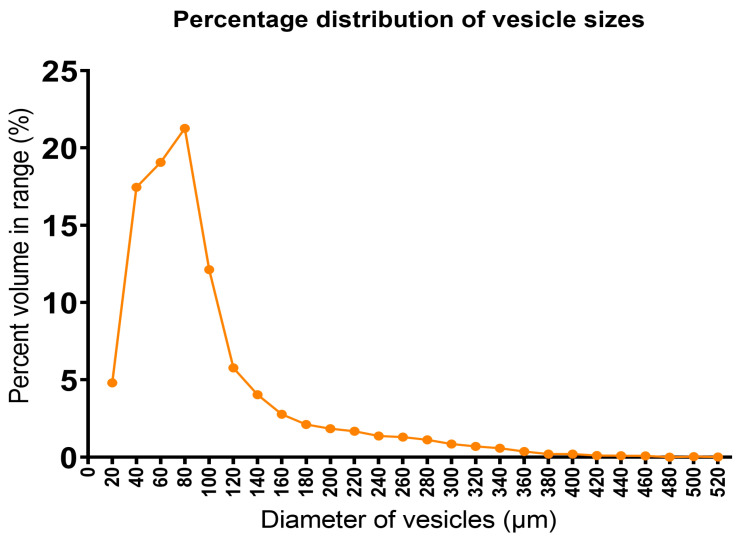
Percentage distribution of vesicle sizes in the foamed S14 SM10% formulation.

Moreover, as shown in Figure 5, the majority (21, 27%) of the vesicles formed by the air dispersed in the matrix has a diameter of 80 microns. It can also be stated that the number of vesicles with a diameter above 120 microns is approximately 25% of the total number.

### 3.8. Differential Scanning Calorimetry

**Figure 6:**
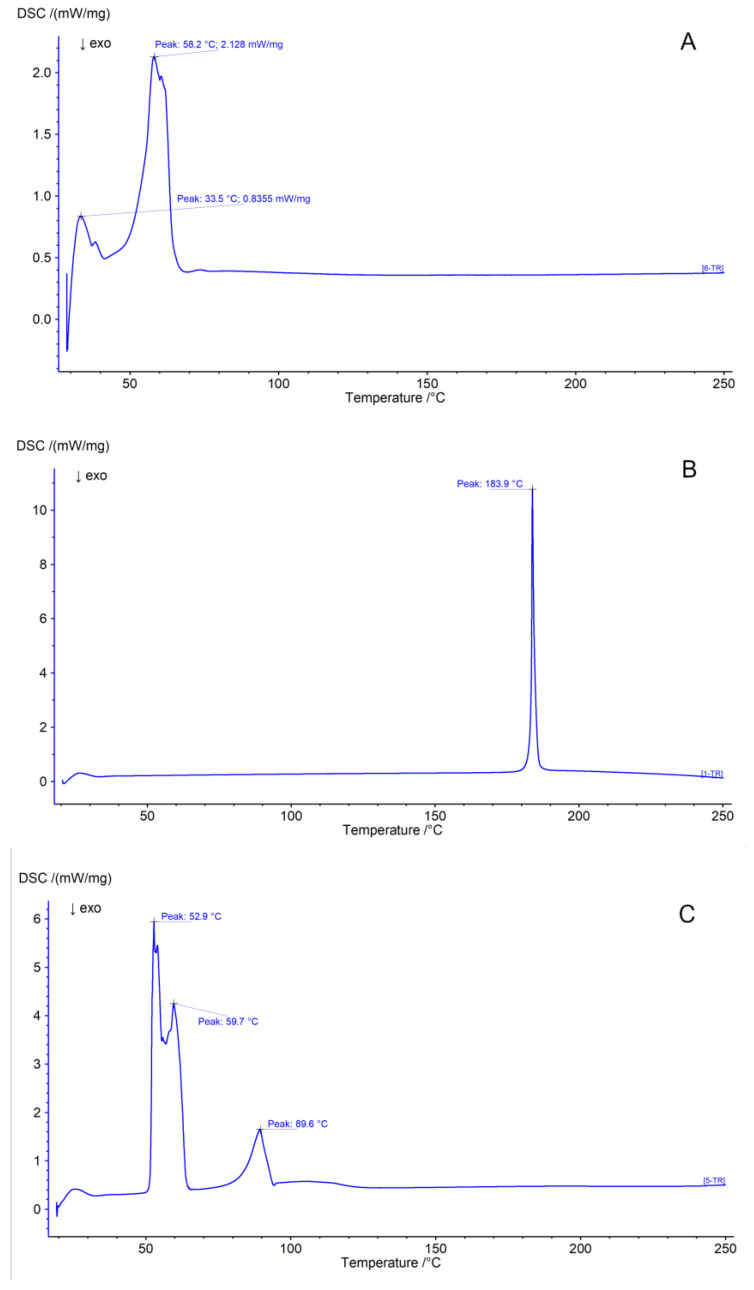

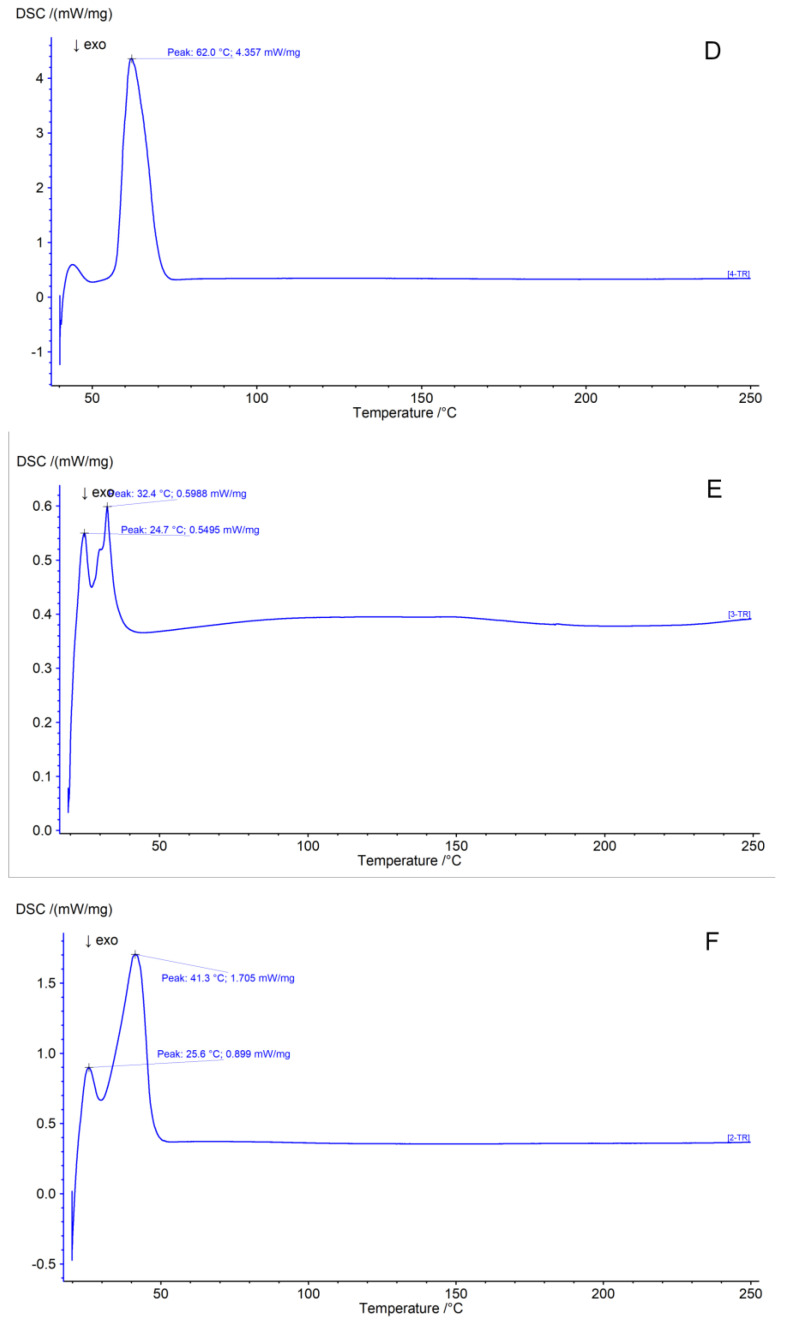
The DSC curves of the materials were obtained in the following order: S14 SM10% formulation (**A**), curcumin (**B**), octadecylamine (**C**), PEG 6000 (**D**), Kolliphor RH40 (**E**), and Gelucire 44/14 (**F**).

The results of the tests are shown in Figure 6. The acquired graphs were compared with the findings of various other researchers. Our high-purity crystalline CUR showed an endothermic peak at 183.9 °C, which can be associated with its melting [53]. The thermal transition of Gelucire 44/14 at 41.3 °C can be considered the melting of the excipient, which starts around 25 °C and ends at 51 °C [54]. Kolliphor RH40 was also tested; however, because the material is semi-solid or partially molten even at room temperature, the complete melting of the excipient was hardly recorded, as our DSC equipment could only start the measurements from ambient temperature. PEG 6000 presented a well-detectable melting with a peak at 62 °C [55], while stearylamine showed complex melting behavior with two peaks due to degradation by atmospheric CO_2_ [56].

### 3.9. Results of Cytotoxicity Assays

As mentioned in Section 2.10, MTT and Neutral Red assays were used to determine the cytotoxicity (and, by extension, the safety of oral application) of the chosen formulation. Dilutions of the test solution (1 g of formulation dissolved in 900 mL PBS) were prepared in 2×, 5×, 10×, 20×, 50×, 100×, and 200× dilution ratios and used to treat the cells.

**Figure 7:**
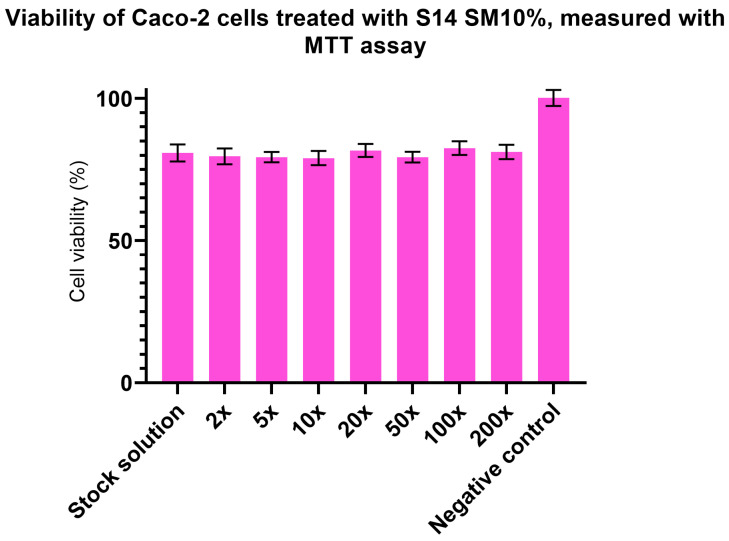
Viability of cells treated with S14 SM10% (compared to negative control) using the MTT staining method.

**Figure 8:**
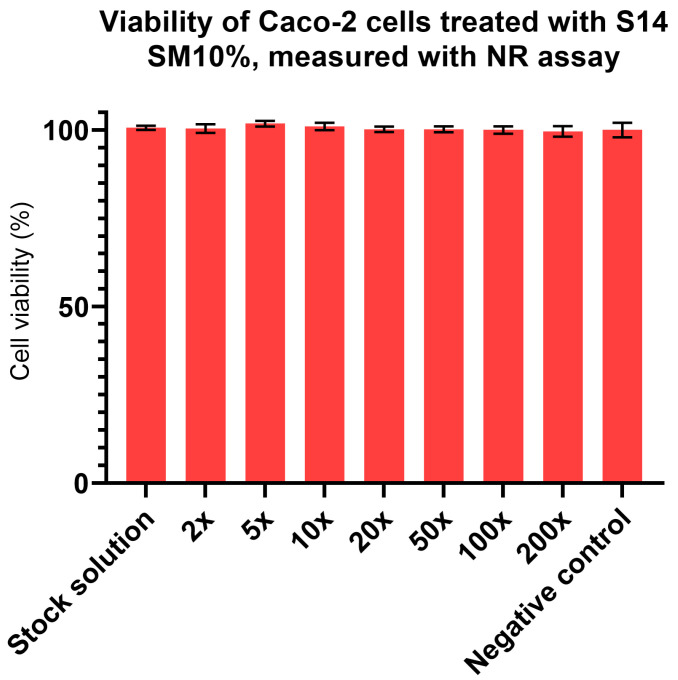
Viability of cells treated with S14 SM10% (compared to the negative control) using the NR staining method.

As shown in Figure 7 and Figure 8, there was no difference in cell viability among the samples. In the Neutral Red assay, all formulations had ~100% cell viability, whereas the results of the MTT assay were 20% lower. This can be explained by the different molecular mechanisms of the assays, as the MTT assay is directly linked to the redox capacity of the mitochondria, while the Neutral Red assay is influenced by the uptake of the stain through lysosomes. As the cytotoxic actions of different groups of chemicals differ from each other, we employed both assays to minimalize false-positive or false-negative results, based on the different sensitivities of the methods [57,58]. Overall, it can be assumed, based on these in vitro experiments, that our compounds have low cytotoxicity and that cytotoxic action mainly affects the mitochondria.

### 3.10. Cellular Transportation Test

**Figure 9:**
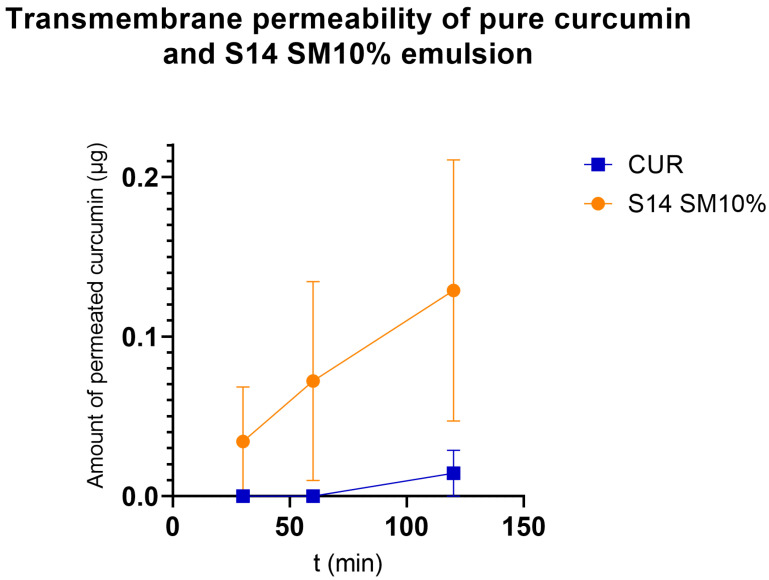
Amount of curcumin permeated through Caco-2 cell monolayers.

Caco-2 cells were seeded on cell culture inserts and grown until a stable cellular monolayer was formed. We compared the cellular transport of pure curcumin with that of our developed formulation. The results are shown in Figure 9. At the first two sampling times, curcumin was not detected in the lower compartment. Compared to pure curcumin, the P_app_ value of curcumin transported in S14 SM10% was approximately ten times greater (1.5216 × 10^−6^) than that of pure curcumin (1.7009 × 10^−7^).

## 4. Discussion

Our aim was to create a gastroretentive self-emulsifying drug delivery system using curcumin as a model API. First, 20 solid formulations were created (Table 1), which were screened for the particle size distribution of the emulsions that could be created through dissolution. After checking the optimal viscosity, dissolution retardants were added, and only those formulations were further tested in which the quality and quantity of the excipient was optimal. Following the comparative dissolution test of S15 SM10% and S14 SM10%, only the latter was further developed using our previously published melt-foaming technology.

PEG 4000 and PEG 6000 were selected as the hydrophilic carriers. They are widely used as water-soluble carriers for water-insoluble drugs [59,60] and as co-solvents to aid the dissolution of CUR in the solid lipid matrix [20,61]. During the DLS analyses, we acquired the size and polydispersity index of the aqueous dispersions of several compositions, and the acquired polydispersity indices were similar to those presented in previous research articles [62,63]. Based on the results of the DLS analysis (Table 2), it can be observed that Gelucire 48/16 provided the smallest hydrated particles dispersed in an aqueous medium. Surprisingly, Gelucire 44/14 was also able to provide one formulation with adequately small droplets, namely S14. Examining the compositions and their particle sizes side by side, it can be observed that Gelucire 48/16 was more potent in creating nanoemulsions with small particles in formulations with a higher Gelucire and Kolliphor RH40 content (compared to the amount of PEG), whereas Gelucire 44/14 was more successful in creating smaller particles in compositions containing more polyethylene glycol. It can also be stated that the types of used polyethylene glycol derivatives did not have a significant effect on the size of the dispersed particles. No articles on this phenomenon were found in the literature. This makes our research novel. This could be explained by the hydrophilicity or hydrophobicity of the API, the HLB value of the tenside, or the placement of API molecules within the micelles of the tenside. We presumed that more hydrophobic APIs would be better encapsulated within a surfactant with a lower HLB value. The size of the micelles also increased when long-chained fatty acid esters were used as emulsifiers. We set the acceptance criteria to a strict 32 nm for particle size and the upper limit of PDI to 0.3 for our samples. We assumed that the dissolution retardants would alter the average size and distribution of the dispersed particles; more precisely, they would increase the average size of the micelles. Thus, we decided to keep the best candidates and eliminate those that did not perform well enough.

Regarding the apparent viscosity values of the molten formulations, no formulations were discarded; however, it was determined that to achieve an ideal texture, the freezing point of the formulation should fall between 47 and 49 °C. In contrast, PEGs increased the viscosity of the molten formulations, as shown in Figure 1. The tested S7 formulation contained only 23.5% PEG 4000, whereas S18 contained 51.8% PEG 6000. When comparing S17 and S18, which contained the same amount of PEG 4000 or 6000 (51.7%), the viscosity values at 51 °C were different, namely 0.382 and 0.63 Pa·s. When comparing S11 and S15 (containing the same PEG 4000 but at different levels, namely 33% and 42.4%), the viscosity values at 51 °C were found to be 0.374 and 0.393 Pa·s, respectively. This indicates that increasing the amount of PEG 4000 slightly increases the viscosity; therefore, the chain length is more important than the polymer ratio in the formulation.

The next step in the development process was to add dissolution retardants to the composition. All three compounds, glycerol distearate (GS; Precirol ATO 5), stearic acid (SA; type 50), and stearylamine (SM), are well-established excipients in the field of pharmaceutical technology [64,65,66]. The particle sizes following the addition of dissolution retardants showed that although the increase in size was significant in all cases, the compositions containing stearylamine provided the favorable, smallest droplets in an aqueous medium. The most stable systems with adequately small particle sizes were acquired using 10 m/m% stearylamine. The correlation between the particle size and the amount of dissolution retardant was nonlinear. However, although the z-average values were acceptable, the high PDI values must be addressed in future research to improve the kinetic stability [62,67].

To find a suitable formulation for extended release, we examined the sample with the closest composition to S15. This formulation was S14, with PEG 6000 instead of 4000 and Gelucire 44/14 instead of 48/16. The ratio of the related components in this one was the same as that in S15. Although S14 almost does not fit the criteria regarding the particle size and polydispersity index, it still produced the third-smallest particles when 10% SM was added to the formulation. The only formulations that produced smaller vesicles were S15 and S8; however, S8 was discarded because of its soft and malleable consistency during the viscosity measurements. S15 SM10% released nearly 90% of its API content in 3 h, similar to first-order release kinetics. This was disadvantageous for us, since such a release profile indicates a tendency for dose dumping. However, the slower drug release of S14 SM10% would contribute to a more balanced drug release, with a well-predictable drug release profile. The S14 SM10% formulation required 5 h to release 55% of its curcumin content. The ICH guidelines declare that three sampling points, roughly at 20–30%, ~50%, and 80% of API release, should be used when evaluating the dissolution test of modified release products [68]. Based on this, it can be stated that although both formulations have a first-order API release kinetic, S14 SM10% has more extended release, making it suitable for formulating sustained-release delivery systems [69]. The prolonged release of S14 can be explained by the results of Da Fonseca Antunes et al., who found that Gelucire 44/14 tends to slow drug dissolution, which can only be overcome by granulation and disintengration-promoting agents [70]. This is further proved by Kim et al., who found that compared to other excipients, Gelucire 44/14 decreased dissolution rate [71].

We used our previously published method to formulate a hot-melt foam system to ensure gastroretentive properties of the system. Micro-computed tomography investigation verified a homogeneous internal structure, as the majority of the air vesicles had a diameter of 80 µm and the vesicles had a narrow size range. Thus, we were able to conclude that the structure was homogenous. The density was calculated using the previously determined volume of the cavities (1.027 mL). Fifteen intact samples were demolded and measured, and each had a density lower than 0.99336 g/mL, with the density of water at 37 °C. This was further confirmed by flotation tests using three random samples in 900 mL of 37 °C artificial gastric juice; all three samples floated on top of the medium.

All the components, as well as the S14 SM10% formulation, were examined using differential scanning calorimetry (Figure 6A–F) to examine the changes in state or chemical structure (e.g., recrystallization, decomposition, oxidation, and loss of moisture). The main peak of curcumin at 183.9 °C cannot be detected in the calorimetric curve of the final formulation, indicating the dissolution or amorphous state of curcumin in the sample, which is in correlation with the literature results of similar compositions [72,73]. This was further verified by PXRD (Figure 3). This revealed a shift in the characteristic peak of curcumin. During API release, once emulsion droplets are formed, the release of the contained curcumin will be much quicker than that of crystalline-state curcumin due to the energy needed to hydrate the crystal lattices, and non-crystalline forms are able to circumvent solvation [74].

In the cytotoxicity assays, a serial dilution was tested on human colorectal Caco-2 cells. Neutral Red assays revealed no harmful effects on the cells (Figure 8). MTT assay results showed only ~80% cell viability (Figure 7). If the given chemical does not cause immediate apoptosis or necrosis in the cell owing to its cytotoxic mechanism but rather inhibits certain cellular functions, the Neutral Red assay, based on passive diffusion, and the MTT assay, which is based on active metabolism, can have altering results [75,76].

After evaluating the low cytotoxic potential of our samples, we compared pure curcumin to our formulation using a well-established in vitro model of intestinal absorption [49]. The results proved that using a self-emulsifying system, even in the presence of dissolution retardants, enhanced curcumin permeability from the upper to the lower chamber, ~10 times greater than that of curcumin alone (Figure 9). Morakul et al. reported increased antioxidant activities in Caco-2 cells when treated with an apigenin-loaded SNEDDS with Gelucire 44/14, Tween 80, and PEG 400, which proves that multiple APIs can benefit from such systems [67].

## 5. Conclusions

In summary, we created a solid self-emulsifying system using Stearylamine, Kolliphor RH40, Gelucire 44/14, and PEG 6000 for the extended release of curcumin as a model API. The solid dispersion was foamed at atmospheric pressure using our batch technology, resulting in a unique, novel combination of solid SEDDSs and targeted gastroretentive systems. Physicochemical investigations confirmed that the API was dissolved in the foamed matrix, while drug dissolution experiments confirmed extended release. In vitro investigations on human Caco-2 cells confirmed a high degree of biocompatibility and enhanced transmembrane permeability.

## Figures and Tables

**Table 1:** Compositions of CUR containing solid SEDDSs ^1^.

Sample	Gelucire 44/14	Gelucire 48/16	Kolliphor RH40	PEG 4000	PEG 6000	CUR
S1	4375	0	4375	1500	0	375
S2	4375	0	4375	0	1500	375
S3	3875	0	3875	2500	0	375
S4	3875	0	3875	0	2500	375
S5	0	4375	4375	1500	0	375
S6	0	4375	4375	0	1500	375
S7	0	3875	3875	2500	0	375
S8	0	3875	3875	0	2500	375
S9	3375	0	3375	3500	0	375
S10	3375	0	3375	0	3500	375
S11	0	3375	3375	3500	0	375
S12	0	3375	3375	0	3500	375
S13	2875	0	2875	4500	0	375
S14	2875	0	2875	0	4500	375
S15	0	2875	2875	4500	0	375
S16	0	2875	2875	0	4500	375
S17	2375	0	2375	5500	0	375
S18	2375	0	2375	0	5500	375
S19	0	2375	2375	5500	0	375
S20	0	2375	2375	0	5500	375

^1^ All numbers represent the amounts of materials in mg.

**Table 2:** Droplet size distribution of the SEDDS composition aqueous phase.

Sample	z Average ^1^	z SD ^1^	PDI Average ^2^	PDI SD ^2^
S1	880.3	112.5	0.823	0.086
S2	948.2	158	0.846	0.091
S3	1369	1292	0.828	0.149
S4	851.2	87.5	0.929	0.09
S5	26.31	22.61	0.154	0.034
S6	13.17	0.032	0.070	0.020
S7	13.08	0.037	0.053	0.006
S8	13.7	0.034	0.161	0.004
S9	504.7	43.64	0.584	0.016
S10	285.6	28.82	0.545	0.082
S11	24.61	19.28	0.162	0.031
S12	67.31	39.81	0.216	0.042
S13	103.7	81.97	0.290	0.035
S14	31.96	25.1	0.300	0.120
S15	15.85	0.19	0.259	0.036
S16	45.01	26.07	0.296	0.077
S17	20.02	8.156	0.185	0.052
S18	14.62	0.12	0.140	0.023
S19	35.2	13.84	0.290	0.097
S20	34.2	17.95	0.329	0.099

^1^ z average is given in nm unit. ^2^ The PDI is a dimensionless quantity between 0 and 1.

**Table 3:** Droplet size distribution of SEDDSs with different dissolution retardants.

Sample	z Average ^1^	z SD ^1^	PDI Average ^2^	PDI SD ^2^
5% SA				
S5	3285	177.5	0.983	0.06
S6	3128	663.8	1.000	0
S7	2298	280.3	1.000	0
S8	3369	1668	0.992	0.014
S11	4857	1439	1.000	0
S14	7047	3873	0.744	0.296
S15	4065	1426	0.998	0.02
S17	6178	515.2	0.953	0.082
S18	5908	1153	0.782	0.193
5% GS				
S5	1038	73.38	0.796	0.039
S6	1063	290.8	0.983	0.120
S7	1801	331.9	0.886	0.103
S8	1797	547.4	0.822	0.168
S11	1304	293.9	0.819	0.114
S14	3195	292.1	1.000	0
S15	2618	1134	0.913	0.151
S17	3407	1673	0.908	0.158
S18	3772	3035	0.944	0.097
5% SM				
S5	1706	741.9	0.687	0.116
S6	855.2	50.22	0.784	0.066
S7	909.8	219.6	0.824	0.117
S8	1074	200.9	0.817	0.023
S11	1185	66	0.701	0.256
S14	2687	521.8	1.000	0
S15	1203	254.8	0.732	0.070
S17	2290	973.4	0.949	0.088
S18	3663	1264	0.891	0.789

^1^ z average is given in nm unit. ^2^ The PDI is a dimensionless quantity between 0 and 1.

**Table 4:** Particle sizes of formulations containing 5 and 10% stearylamine, based on DLS measurements.

Sample	z Average ^1^	z SD ^1^	PDI Average ^2^	PDI SD ^2^
5% SM				
S5	1197	108.1	0.893	0.116
S6	855.2	50.22	0.784	0.066
S7	909.8	219.6	0.824	0.117
S8	1074	200.9	0.817	0.023
S11	1185	66	0.701	0.256
S14	2687	521.8	1.000	0
S15	1203	254.8	0.732	0.070
S17	2290	973.4	0.949	0.088
S18	3663	1264	0.891	0.789
10% SM				
S5	3709	310.2	0.453	0.358
S6	2539	108.9	0.995	0.008
S7	6384	919.9	1	0
S8	2391	101.4	0.875	0.031
S11	4823	631.0	1	0
S14	2122	172.0	0.883	0.283
S15	666.6	155.2	0.633	0.094
S17	3209	433.4	1	0
S18	5908	1153	0.782	0.193

^1^ z average is given in nm unit. ^2^ The PDI is a dimensionless quantity between 0 and 1.

**Table 5:** Correlation coefficients of dissolution tests of S14 and S15 with 10% SM content.

	S14 SM10%	S15 SM10%
Zero-order	0.9806	0.9649
First-order	0.9898	0.9893
Korsmeyer-Peppas	0.8745	0.9017

## Data Availability

The original contributions presented in the study are included in the article, further inquiries can be directed to the corresponding author.
